# Challenges and coping strategies of nursing interns in training for nationwide medical technical skills competition: a qualitative descriptive study

**DOI:** 10.1186/s12912-025-03387-0

**Published:** 2025-07-01

**Authors:** Zhongchen Luo, Qing Wang, Jie Li, Hongli Chen, Hong Li, Keyan Xue, Jiao Tang

**Affiliations:** 1 School of Nursing, Guizhou Medical University, Guiyang, China; 2 School of Nursing, Chinese Academy of Medical Sciences & Peking Union Medical College, Beijing, China; 3 School of Nursing, Lanzhou University, Lanzhou, China; 4 Mental Health Center, West China Hospital/West China School of Nursing, Sichuan University, Chengdu, China; 5 Department of Nursing, The Affiliated Hospital of Guizhou Medical University, Guiyang, China; 6 School of Nursing, Chongqing Medical University, Chongqing, China; 7 Department of Nursing, The First Affiliated Hospital of Chongqing Medical University, Chongqing, China

**Keywords:** Nursing interns, Nursing technical skills competition, Challenges, Coping strategy, Descriptive qualitative study

## Abstract

**Background:**

Nursing technical skill competitions have proven to be an effective and efficient method for enhancing both learning and teaching within the field. Investigating the obstacles encountered by participants and their strategies for overcoming these challenges is crucial for refining preparation training strategies and daily pedagogical practices. The aim of this study was to examine the challenges and coping strategies of nursing interns as they prepare for the National Medical Technical Skills Competition for College Students in China.

**Methods:**

A descriptive qualitative study using face-to-face semistructured interviews was conducted.

**Results:**

Identified challenges included (1) managing training workload and recovery time; (2) a lack of knowledge application and integration abilities; (3) inconsistent training standards affecting training progress; (4) cohesion negatively impacted by an adverse team climate; (5) difficulty in balancing training, academics, and job hunting; (6) emotional resilience and stress regulation; and (7) motivation maintenance. Coping strategies encompassed (1) effectively arranging training content and duration, (2) adopting innovative training approaches and teaching methods, (3) aligning the frequency and difficulty of training assessments with students’ current abilities, (4) enhancing the professional practice and teaching skills of trainers, and (5) pre-selecting contestants based on their characteristics and professional capacity.

**Conclusion:**

Nursing interns encounter numerous challenges while preparing for the national medical skills competition. It is crucial to create a well-organized training schedule, address negative emotions, provide timely psychological support and establish a high-caliber competition training team to enhancing training effectiveness. Additionally, implementing comprehensive reforms in nursing education teaching methodologies is vital for improving the professional competence of student interns, particularly in strengthening their clinical decision-making skills for analyzing and resolving nursing issues in specific clinical settings.

**Clinical trial number:**

Not applicable.

**Supplementary Information:**

The online version contains supplementary material available at 10.1186/s12912-025-03387-0.

## Background

Nursing, as a highly practiced discipline, necessitates that nurses possess exceptional clinical skills, critical thinking, and teamwork. Nursing skills competitions play a crucial role in academic institutions by advancing undergraduate education reform and enhance teaching quality. These competitions serve as a valuable tool for enhancing the quality of nursing training and fostering the core competencies of nursing students by refining practical skills, critical thinking abilities, humanistic care practices, and communication skills [1, 2]. Additionally, it has been proves to be an effective and efficient platform for promoting learning, teaching, fostering interest, expanding knowledge and enhancing collaborative inter-professional practice ability within healthcare professions [3–5].

Since 2010, the Ministry of Education in China has organized national clinical skills competitions for undergraduate medical students. This competition aims to promote comprehensive reform in medical education, innovate practical teaching systems, and enhance the overall quality and technical skills of medical students [6, 7]. Over the years, it has become a highly influential event within the national medical education community, with participation growing from 19 colleges and universities in the first session to approximately 115 institutions in 2021 [8, 9].

The 2021 competition, the 10th in the series, marked a significant milestone by introducing a nursing professional track for the first time. This new track aimed to assess the integrative competence of final-year nursing students in their internship phase. The evaluation focused on core knowledge, essential clinical skills, critical thinking, humanistic care, collaboration, and particularly on nurse-patient communication and health education. The nursing track quickly gained nationwide participation, with over 200 nursing colleges and universities taking part, encompassing nearly all nursing schools in China [11].

The competition was structured in three stages: school-level preliminary rounds, regional contests, and a national championship. The preliminary rounds were held by individual institutions, while the regional and national contests were managed by the Teaching Steering Committee for Nursing Programs of Higher Education Institutions under the Ministry of Education. This competition assessed 40 nursing technical skills and knowledge domains, including fundamental nursing techniques, health assessment, internal medicine, surgical procedures, obstetrics and gynecology, pediatric care, critical care nursing, geriatric nursing, community nursing, and overall professional competencies [10].

The assessment format progresses from individual evaluations in the preliminary rounds to team-based scenarios in the regional and national stages, with increasing complexity and emphasis on clinical reasoning, teamwork, and patient communication. In the preliminary rounds, candidates were assessed individually through case-based scenarios that integrated fundamental clinical reasoning while emphasizing standardization and competency in technical skills. The regional contests adopted a team-based relay format consisting of three interrelated scenarios based on progressively complex clinical cases, aimed at evaluating participants’ ability to integrate theoretical knowledge with practical application, as well as their clinical reasoning skills, teamwork capabilities, and patient communication. The final national championship featured comprehensive clinical scenarios that incorporated situational changes and variations in patient conditions, further assessing candidates’ clinical reasoning, humanistic care, patient communication, as well as team coordination, task division, and flexibility [6, 10].

The nursing track aimed to encourage nursing schools to modernize their teaching methods and improve the quality of nursing education [6, 10]. Evidence suggests that participating in this competition significantly enhances students’ clinical thinking, professional confidence, attitudes, and career development clarity [12, 13]. Moreover, insights from the competition’s training model have been applied to clinical teaching in medical courses, thereby enhancing the overall quality of education [14].

Notably, while the competition was held annually from 2010 to 2021, the nursing track was introduced only in 2021 and has not been held at the national level since then. Nevertheless, the 2021 nursing track remains the largest and most reform-driven national competition in China’s medical education history, providing valuable insights into the future direction of nursing education in China. The impact of this competition continues to resonate at the provincial and university levels, with numerous local competitions adopting similar themes, formats, and objectives. For instance, Gansu Province held its first Medical Technical Skills Competition for College Students in June 2023, while Southwest Medical University successfully held the 2024 Medical Technical Skills Competition (Nursing Professional Track) in November 2024. Additionally, Bengbu Medical College organized the 2024 Medical Technical Skills Competition (Nursing Professional Track) in January 2025). These events reflect the ongoing influence of the 2021 nursing track and its significant role in shaping nursing education and skills training across China.

Qualitative description is a methodology that offers a comprehensive overview of an event using simple and accessile language. This approach involves lower levels of interpretation compared to high-inference qualitative methods such as phenomenology or grounded theory, emphasizing a less conceptual and less abstract rendering of data. It allows researchers to maintain closer proximity to their data, facilitating a more direct understanding of the words and events involved. Consequently, it surpasses many other methods in providing straightforward descriptions of phenomena [15, 16].

Therefore, the study aims to examine the challenges and coping strategies faced by intern nursing students as they undergo preparatory training for the first nursing track of the National Medical Technical Skills Competition for College Students in China utilizing a qualitative descriptive study. Additionally, it seeks to uncover coping strategies from the contestants’ perspectives, providing valuable insights for enhancing the training programs of nursing institutions in relation to nursing technical skills competitions, as well as for improving teaching strategies and incorporating innovative teaching methods.

## Methods

### Design

A qualitative descriptive study was undertaken, employing semistructured interviews. Data analysis was performed using content analysis [15, 16].

### Participants

From March to May 2021, undergraduate nursing interns were recruited from Chongqing Medical University and Guizhou Medical University. These interns had received specialized training in preparation for the nursing track of the National Medical Technical Skills Competition for College Students in China. Inclusion criteria included enrollment and participation in the preparation training for the National Medical Technical Skills Competition for College Students for ≥ 1 month and voluntary participation in this study. Exclusion criteria comprised students who withdrew from the preparation training for personal reasons.

A purposive sampling strategy was employed to recruit research participants, considering factors such as gender, age, family residence, occupational plans, experience in nursing skills competitions, prize type in the National Medical Technical Skills Competition, and training duration. To ensure maximum variation, the sampling was further refined within this purposive framework [17]. The sample size was determined based on information saturation, assessed through ongoing analysis during data collection [18]. As data saturation was gradually reached, 17 students were ultimately invited to participate in the interviews, all of whom agreed and expressed interest in participating. No students were excluded based on the established inclusion or exclusion criteria. General information about the participants is presented in Table 1.

**Table 1:** Demographic characteristics of the participants

Participant	Gender	Age	Family residence	Occupational planning	Experience of nursing skills competitions	Type of prize in the Medical Technical Skills Competition
1	Female	21	City	Nursing teacher	School level competition	Second prize of the Central and South China Division Competition
2	Male	23	Rural area	Nurse or nursing teacher	None	None
3	Male	22	Rural area	Nursing teacher	Both school and university-level	None
4	Female	22	Township	Other professions related to nursing (e.g. medical representative, health manager) or non-nursing related occupations (e.g. civil service)	School level competition	None
5	Female	22	Rural area	Other professions related to nursing (e.g. medical representative, health manager)	University-level competition	None
6	Female	22	Rural area	Nurse	None	Second prize of the Central and South China Division Competition
7	Female	21	Township	Nurse or nursing teacher	None	Second prize of the Central and South China Division Competition
8	Female	21	Township	Nurse or nursing teacher or other professions related to nursing (e.g. medical representative, health manager)	University-level competition	Second prize of the Central and South China Division Competition
9	Female	21	Rural area	Nurse or nursing teacher or other professions related to nursing (e.g. medical representative, health manager)	University-level competition	None
10	Female	22	Rural area	Nurse or other professions related to nursing (e.g. medical representative, health manager)	School level competition	None
11	Male	21	City	Freelance work	None	None
12	Female	23	Rural area	Nurse or other professions related to nursing (e.g. medical representative, health manager)	None	None
13	Female	23	City	Nurse	None	None
14	Female	21	City	Nurse or nursing teacher or other professions related to nursing (e.g. medical representative, health manager)	None	Third prize of the national competition
15	Female	21	City	Nurse or nursing teacher	Municipal competition	Third prize of the national competition
16	Female	23	City	Nurse or non-nursing related occupations (e.g. civil service)	Municipal competition	Third prize of the national competition
17	Female	22	City	Nurse or nursing teacher	None	Third prize of the national competition

### Data collection

Semistructured interviews, which included open-ended questions to allow participants to introduce themes not initially considered, were conducted. The interview outline was developed by reviewing relevant literature and aligning it with the study’s objectives. This outline was subsequently revised by a training supervisor, a Ph.D. student in nursing, and three nursing educators who have been involved in training students for this competition. To finalize the questions and determine the interview duration, three nursing students who met the inclusion and exclusion criteria were pretested using convenience sampling.

Semistructured interviews, featuring open-ended questions to facilitate participants in raising themes not initially considered [19], were conducted. The interview outline was developed by reviewing relevant literature and aligning it with the study’s purpose. Subsequently, it was then revised by a training supervisor, a Ph.D. student in nursing, and three nursing teachers who have been involved in training students for this competition. To finalize the questions and establish the appropriate interview duration (Table 2), we conducted a pretest with three nursing students who met the inclusion and exclusion criteria, selected through convenience sampling.

**Table 2:** Outline of the semi-structured interview

No	Question
1	What difficulties and challenges have you encountered during the training for the National Medical Technical Skills Competition for College Students?
2	What kind of help or support do you expect in coping with the challenges in the training process?
3	What suggestions do you have to improve the feasibility and efficiency of preparation training for the competition?

We acknowledge that the interview guide used in this study partially overlaps with our previous research published in Chinese [12]. The previous study, titled ‘The motivation and growth experience of nursing students participating in the nursing track of the Chinese College Students Medical Skills Competition: a qualitative study’, focused on nursing students’ growth experiences and participation motivations in the National Medical Technical Skills Competition. While our previous work explored themes such as participation motivation and growth experience, the current study specifically investigates the challenges faced by nursing students and their coping strategies. This new focus provides valuable insights into the difficulties encountered by students and their methods for overcoming these obstacles, representing a distinct aspect not covered in our previous publication. The complete interview guide for the current study, which includes questions addressing these new aspects, is available as Supplementary File 1.

Interviews were conducted in empty training rooms or faculty offices of the school/hospital nursing skills training center, ensuring a private and quiet environment. The interviewers (ZCL and JT), who had undergone the “Theory and Practice of Qualitative Research Workshop” at Fudan University, had extensive experience in qualitative research and were actively involved in the training of nursing students. Prior to the interviews, the study’s purpose and key points of cooperation were explained, informed consent was obtained, and assurance was given that interview data would be used solely for academic research. Ethical approval was obtained from the Ethics Committee of Chongqing Medical University (Grant No. 2020013).

During the interviews, participants were encouraged to articulate their ideas using various interview techniques, such as rhetorical questions, follow-up questions, repetition, summaries, and responses, as appropriate. The interviewers fostered a comfortable environment to elicit candid responses. The duration of each interview was unrestricted, allowing participants to fully discuss their experiences and insights. Data collection concluded when no new information emerged, indicating that information saturation had been reached. Interview durations ranged from 45 to 60 min. The collected data were securely stored in encrypted form on private computers managed by ZCL and JT, who were responsible for transcription and analysis, thereby emphasizing the importance of preserving student privacy. The interview recordings were transcribed into text and subsequently returned to participants for verification of content authenticity and accuracy.

### Data analysis

After completing the interviews, audio recordings were transcribed within 24 h. One researcher (ZZH) transcribed the audio data, while another researcher (ZCL) cross-checked the audio against the transcribed interview transcripts. The data were analyzed using content analysis [16]. Two researchers, ZCL and QW, both of whom are nursing faculty with extensive experience in qualitative research, independently reviewed the transcripts to understand the data and identify essential concepts.These key concepts were then utilized to construct a coding framework within Word documents. Subsequently, the researchers systematically coded the remaining transcripts line by line, highlighting significant passages that elucidated the challenges and coping strategies of intern nursing students undergoing preparation training for the National Nursing Skills Competition. Similar codes were clustered together to create categories, which were then organized into themes. Throughout the data analysis process, the two authors, ZCL and QW, convened frequently to collaboratively discuss and reach consensus on the identification and refinement of categories and themes.

### Trustworthiness

The trustworthiness of this study was established through the criteria of credibility, transferability, dependability, and confirmability [20]. The interviewers had undergone qualitative research training and possessed extensive experience in this field. The representativeness of the research subjects was a key consideration during participant selection. Interviews were conducted in separate rooms to ensure a smooth process. To enhance the reliability, validity, and rigor of the analysis, two researchers independently analyzed the data, compared their results, discussed and resolved discrepancies, and returned the transcripts and analysis results to the participants for confirmation. Additionally, the Criteria for Reporting Qualitative Research (COREQ) was employed to assess the quality of the study [21].

## Results

Out of the 17 undergraduate nursing interns, 14 were female and aged between 21 and 23 years. 41% of their families lived in urban regions, 41% in rural areas, and 18% in townships. Additionally, 53% of the individuals had privous experience on nursing skills competition at the college, school, or municipal level. Furthermore, four interns secured second prize in the Central and South China Division nursing skills competition, while another four attained third prize in the national Medical Technical Skills Competition.

Challenges faced by nursing students in the preparation training were identified through the identification of seven themes:1) managing training workload and recovery time; 2) a lack of knowledge application and integration abilities; 3) inconsistent training standards affecting training progress; 4) cohesion negatively impacted by an adverse team climate; 5) difficulty in balancing training, academics, and job hunting; 6) emotional resilience and stress regulation and 7) motivation maintenance.

Additionally, coping strategies for challenges encountered in the training program were identified through the analysis of five key themes: (1) effectively arranging training content and duration, (2) adopting innovative training approaches and teaching methods, (3) aligning the frequency and difficulty of training assessments with students’ current abilities, (4) enhancing the professional practice and teaching skills of trainers, and (5) pre-selecting contestants based on their characteristics and professional capacity (Table 3).

**Table 3:** Summary of themes and sub-themes

Theme	Sub-theme
Challenges of nursing students in preparation training for the competition	Managing training workload and recovery time
	A lack of knowledge application and integration abilities
	Inconsistent training standards affecting training progress
	Cohesion negatively impacted by an adverse team climate
	Difficulty in balancing training, academics, and job hunting
	Emotional resilience and stress regulation
	Motivation maintenance
Coping strategies to challenges in the training program	Effectively arranging training content and duration
	Adopting innovative training approaches and teaching methods
	Aligning the frequency and difficulty of training assessments with students’ current abilities
	Enhancing the professional practice and teaching skills of trainers
	Pre-selecting contestants based on their characteristics and professional capacity

### Challenges of nursing students in Preparation training for the competition

#### Theme 1: managing training workload and recovery time

During the training process, appropriate rest and reflection time are crucial for optimal learning outcomes and students’ emotional well-being. However, student feedback indicates that the training schedule is excessively intensive and protracted, impeding their ability to obtain adequate rest and recuperation. Prolonged periods of high-intensity training can lead to the accumulation of fatigue, which adversely affects both cognitive performance and overall well-being. Moreover, the excessively frequent training regimen results in a lack of sufficient time for pupils to engage in profound contemplation and independent practice, both of which are essential for comprehending and consolidating new acquired skills. Insufficient time may lead to decreased training effectiveness and unfavorable learning outcomes.

*“The total training schedule is extensive*,* allowing for only one day off per week. It requires additional night practice and longer training sessions without rest periods. Furthermore*,* as this is our inaugural competition nursing program*,* frequent disagreements arise between clinical instructors and academic faculty*,* leading to a lack of consistency in operational standards on a daily basis. This inconsistency causes significant distress among students*,* who perceive the operations as ineffective”* (Student 16).

*“I find it difficult to read at night due to an overactive mind. The training is excessively intensive*,* providing little time for reflection and practice*,* which renders the training inefficient.”* (Student 4).

#### Theme 2: A lack of knowledge application and integration abilities

They noted that combining academic knowledge with practical application poses significant challenges. Memorizing protocols and standardized procedures does not adequately represent the essence of humanistic care. Moreover, as competition becomes increasingly clinical in nature, a patient-centered approach is essential; however, the need to enhance operational efficiency may lead to the neglect of critical nuances and the development of clinical reasoning. During the training process, steps are typically executed according to scoring criteria, resembling the conduct of exercises, with a focus on assessing symptoms and indications rather than fostering comprehensive clinical thinking.

*“Humanistic care transcends mere memorization or the mechanical standardization of processes; such an approach leads to a ‘copy and paste’ methodology that fails to embody the essence of humanistic care. "* (Student 10).

*“The current competitive model is predominantly clinical and patient-centered*,* prioritizing the saving of patients. However*,* the emphasis on operating more quickly often results in the neglect of critical details and clinical reasoning.”* (Student 17).

*“The training rigorously follows scoring criteria*,* which can result in a rigid format that resembles answering questions. Consequently*,* reading cases sometimes feels akin to taking a test*,* relying heavily on symptoms and signs for judgments*,* without significantly enhancing clinical thinking.”* (Student 13).

#### Theme 3: inconsistent training standards affecting training progress

Some students report a lack of efficient communication and coordination between clinical and school instructors during the training process, which leads to frequent changes in operational norms. This instability not only affects students’ learning outcomes but also contributes to feelings of demotivation and ineffectiveness in their training operations. Furthermore, team members tend to focus excessively on detailed discussions during the training process, neglecting time management and efficiency optimization. As a result, valuable training time is not utilized effectively in more critical learning areas. This inefficiency not only hinders students’ overall learning progress but may also reduce their comprehension and understanding of the learning content.

*“Individuals sometimes become entangled in minor tasks*,* dedicating entire days to exploration. We should optimize our time usage to focus on more beneficial activities. In terms of theoretical knowledge*,* individual self-study efforts should be encouraged*,* while the teacher’s role could prioritize case studies and practical applications*,* allocating time judiciously.”* (Student 17).

*“The scheduling cycle is somewhat condensed; new content can only be practiced in the evenings or at noon. This intensive schedule leaves little time for adequate reflection.”* (Student 2).

#### Theme 4: cohesion negatively impacted by an adverse team climate

During preparation for the nursing skills competition, the enthusiasm and emotional state of each team member significantly impact the overall performance. Some participants noted that if individual members of the team lack enthusiasm, it can adversely affect the morale of the entire group; observing teammates with concerns or in a negative emotional state can diminish the training atmosphere and reduce efficiency. Even if one member’s personal situation is strong, it cannot fully compensate for the poor morale of the entire team. It is evident that the overall emotional state of the team is crucial; thus, it requires concerted efforts to ensure that every member is in high spirits.

*“Peer influence plays a significant role in team dynamics; when certain team members lack motivation*,* it adversely affects the overall enthusiasm of the group. I experienced a period of fatigue and diminished motivation during the middle of the training week*,* and the lack of motivation among my peers further dampened the training atmosphere. However*,* after making adjustments*,* I observed that as my mood improved*,* it positively influenced the overall morale of my classmates*,* highlighting the impact that an individual can have on the team’s mood. "* (Student 16).

*“Sometimes*,* observing fellow students who are concerned or in low spirits can contribute to a subdued training atmosphere*,* ultimately reducing overall efficiency. In a team setting*,* individual motivation alone is insufficient; the collective well-being of all members is crucial for fostering a positive and effective training environment.”* (Student 17).

#### Theme 5: difficulty in balancing training, academics, and job hunting

The competitions are designed for nursing undergraduates currently engaged in clinical internships, with training sessions scheduled for the second semester of their final year before graduation. This period is crucial as it coincides with the recruitment season for soon-to-be graduates and the preparation for graduate school entrance exams. Consequently, students often encounter challenges in balancing competition training, academic responsibilities, and job hunting, which can adversely affect their overall performance and increase stress levels.Additionally, students preparing for competitions must navigate the complexities of job hunting, postgraduate entrance exams, and other significant decisions during this time. Many participants struggle with time management, as training demands a substantial portion of their schedule, leaving insufficient time for job searches or nursing exam preparations. Furthermore, the extensive time commitment required for training can result in diminished energy and time to actively pursue employment opportunities. By the time the competition concludes, some prime job opportunities may have already been missed. This concern over potential risks can hinder students’ focus on competition preparation and may negatively impact the overall effectiveness of team training.

*“Participating in training leaves little time to search for employment and prepare for nursing exams*,* creating a sense of stagnation in both areas.”* (Student 6).

*“The extensive duration of training consumes the majority of our time*,* resulting in insufficient energy and opportunity to pursue job openings during this critical period for job searches. Consequently*,* by the time we attempt to secure a suitable position following the competition*,* we risk missing out on potentially valuable opportunities altogether.”* (Student 15).

*“The preparation period for the competition poses challenges in simultaneously seeking employment*,* preparing for graduate studies*,* and managing transfers to different graduate programs. We find ourselves at a crossroads*,* weighing various considerations. While the competition is significant*,* it is not our only option. The efficiency of the entire team is compromised when each member shares these concerns.”* (Student 17).

#### Theme 6: emotional resilience and stress regulation

When some students encounter skill bottlenecks during training, they often experience anxiety and unease, which can manifest as feelings of inadequacy and fear regarding an uncertain future. This psychological condition may hinder learning progress and negatively affect overall performance, leading to feelings of inferiority and diminished self-confidence. Furthermore, this psychological stress can impact an individual’s motivation to learn and their performance in competitions. Additionally, persistent criticism and correction during training may elicit fear and avoidance responses, potentially suppressing the desire to learn and diminishing initiative. These stress-induced negative emotions can adversely affect students’ physical health and adaptability, resulting in decreased immunity and impaired sleep quality.

*“Sometimes we worry that our performance in the competition might affect the reputation of our school. We fear that our performance could bring negative consequences to the school*,* which also increases our psychological pressure…….This has led to a collective sense of apprehension among the students*,* and those who find it challenging to adapt may exhibit diminished resilience*,* as evidenced by reports of colds and disrupted sleep among several individuals.”* (Student 2).

*“I consistently struggle during practice sessions*,* feeling as though I have reached a bottleneck where I am uncertain of where to position my hands and feet. Nightmares plague me nightly*,* filled with the anxiety of not being selected. I often question why I perform poorly while others excel*,* and these thoughts occupy my mind incessantly. "* (Student 4).

*“When I am repeatedly corrected*,* fear begins to set in*,* leading to hesitation in my actions. This apprehension about making mistakes can result in avoidance or reluctance to proceed*,* ultimately disrupting the effectiveness of the training.”* (Student 16).

#### Theme 7: motivation maintenance

Some students demonstrate a lack of motivation and a clear sense of purpose during their competition preparation, leading to diminished enthusiasm and even negative emotions. This absence of motivation and the negative feelings encountered during training may arise from their incentive to compete being driven not by a desire to achieve optimal results, but rather by a wish to evade other responsibilities, such as clinical internships. Many students believe that their current abilities are insufficient to secure any awards, which fosters a reluctance to fully engage in the process for what they perceive as a remote possibility of success, resulting in a tendency to maintain a ‘low-energy state.‘. Some students reported:

*“At the onset of the training process*,* my motivation was not particularly strong*,* and I experienced moments of uncertainty regarding my purpose*,* which left me in a state of detachment. Despite my best efforts and a desire to excel*,* I struggled to maintain consistent engagement. There were instances when I felt quite lax*,* accompanied by occasional periods of heightened despondency.”* (Student 10).

*" I joined primarily to step away from clinical practice*,* never considering the possibility of winning a prize. Given my current capabilities*,* I believe that winning is not feasible*,* and I am reluctant to invest all my energy into this slim prospect. Currently*,* I find myself in a state of conserving energy.”* (Student 11).

***“****When certain team members lack motivation*,* it adversely affects the team’s enthusiasm. I experienced a period of fatigue and diminished motivation during the middle of the training week*,* and the lack of drive among my peers further dampened the training atmosphere. Upon making adjustments*,* I noticed that as my mood improved*,* it positively influenced the overall morale of my classmates*,* underscoring the impact of an individual on the team’s mood.”* (Student 16).

*“At times*,* observing fellow students who are concerned or in low spirits can create a subdued training atmosphere*,* ultimately reducing overall efficiency. In a team*,* individual motivation alone is insufficient; the collective well-being of all members is crucial for fostering a positive and effective training environment.”* (Student 17).

### Coping strategies to challenges in the training program

#### Theme 1: effectively arranging training content and duration

Participants raised several concerns regarding training strategies and scheduling. These issues encompass the need to establish clear training goals for each phase, allocate time effectively, implement a progressive training model, plan training sessions in advance, and maintain flexibility throughout the training period. It is essential to formulate specific training objectives and plans for different phases, emphasizing the mastery of key concepts and challenging aspects of training, as well as the progressive development of students’ abilities. Furthermore, given that students experience pressures from exams and employment during the training period, time management should consider their overall well-being. The training duration may be extended appropriately to ensure that the content is organized pragmatically, to effectively address special situations, and to mitigate excessive stress and fatigue that could hinder training effectiveness.

*“It is essential to articulate clear training objectives*,* whether the emphasis is on developing operational skills*,* enhancing critical thinking abilities*,* or striving for excellence. A focused approach is vital. "* (Student 10).

*“Consider commencing with one or two months of dedicated training on a single operation*,* followed by case studies*,* and concluding with collaborative refinement among team members. This layered training approach may yield superior results.”* (Student 16).

*“The middle and later stages of training present unique challenges*,* including pressures from examinations and employment concerns. Consequently*,* a more flexible schedule is recommended during this period. Overly rigid scheduling can lead to fatigue*,* potentially undermining the effectiveness of the training.”* (Student 15).

#### Theme 2: adopting innovative training approaches and teaching methods

Participants indicated that observing competition videos from other schools and learning from their experiences can aid in the development of more efficient and specific operational procedures. By comparing the operational procedures of various schools, they can gain insights into the strengths and experiences of others, which, when integrated with their own context, allows for the creation of procedures that better meet their needs. Furthermore, it is advisable to adopt the practice of reviewing competition videos from other schools to leverage their experiences in crafting faster and more precise operational procedures, as well as to utilize video resources and operational templates to enhance participants’ skills and readiness for competition.

*“We attended various competitions*,* recording videos to observe and analyze the operational processes employed by other schools. This experience proved invaluable*,* enabling us to design and refine our operation processes based on a synthesis of our experiences and those of other institutions.”* (Student 16).

*“I have come to understand that certain majors meticulously document every operation according to the most comprehensive and standardized procedures*,* creating a video resource for subsequent classes after each student cohort participates in the competition. Alternatively*,* a condensed video template*,* lasting 6 to 8 minutes*,* could be developed for competition operations*,* thereby simplifying the learning process for the next group of students who can follow the established template.”* (Student 14).

#### Theme 3: aligning the frequency and difficulty of training assessments with students’ current abilities

Students indicated that the daily training schedule should prioritize preparation for assessments, specifically focusing on the frequency and difficulty level of these evaluations. Additionally, the frequency and difficulty of training assessments should be aligned with the participants’ current abilities. This approach would facilitate a more effective transition for students into the competitive environment, thereby enhancing the overall effectiveness of the training program.

*“Incorporating regular simulation competition examinations tailored to the ability levels of participants*,* where evaluations are conducted based on specific cases*,* serves to enhance psychological resilience for all involved.”* (Student 3).

*“Conducting assessments with a more formalized structure*,* as opposed to a casual approach*,* fosters a sense of ritual among students. This increased sense of focus contributes to greater efficiency.”* (Student 12).

*“The test content should be designed to present a slightly higher level of difficulty compared to routine training. This strategy encourages greater effort and critical thinking in subsequent training sessions. While it is important to introduce challenges*,* the emphasis should remain on progressing through levels without excessively increasing the difficulty. "* (Student 16).

#### Theme 4: enhancing the professional practice and teaching skills of trainers

Students have reported that the personality traits of training instructors, their instructional methods, and communication skills significantly influence training effectiveness. Traits such as being excessively lively or overly introverted can impact students’ learning states and emotions, which in turn affects their ability to complete practical training exercises. Furthermore, students have highlighted the importance of the tone and choice of words used by instructors during guidance. Excessively harsh or severe criticism can have a long-lasting negative impact on students; therefore, it is essential to consider and respect others’ feelings. Additionally, students have suggested that instructors should provide feedback after tasks are completed and minimize interruptions during activities. This approach can help students clarify their thoughts, reduce tension, and enhance learning efficiency. Clearly, focusing on the selection and skill development of instructors can significantly improve the effectiveness of competition preparation training.

*“The teacher’s personality significantly influences the effectiveness of training. An overly lively teacher can foster a cheerful atmosphere; however*,* this may result in a more relaxed mood that undermines the quality of operational training tasks. Conversely*,* an introverted teacher may contribute to a somewhat subdued training environment*,* which could lead to students feeling less motivated to complete the day’s assignments.”* (Student 16).

*“Some teachers may express dissatisfaction with students’ mistakes in a manner that is perceived as harsh*,* often without realizing the lasting impact of their tone and language. It is crucial*,* whether interacting with teachers or peers*,* to avoid overly harsh language and to be mindful of others’ feelings.”* (Student 5).

*“I prefer that teachers provide feedback after the completion of our operations without interrupting the process. Frequent interruptions can disrupt my thought process and create confusion in my thinking. After a systematic review by the teacher*,* it is beneficial to allow students a few minutes to reflect before immediately redoing the operation. This brief reflection period aids in organizing thoughts and enhancing overall performance.”* (Student 4).

#### Theme 5: Pre-selecting contestants based on their characteristics and professional capacity

Students suggest that the selection process for future contestants should consider both hard and soft skills. Specifically, final-year students who have already secured admission to graduate programs or received confirmed job offers may be particularly well-suited for participation. These students not only demonstrate strong academic and professional competencies but also face less pressure related to further education or job hunting, allowing them to dedicate more time and energy to competition preparation. Alternatively, lower-year students could be identified in advance for targeted training, allowing them ample time for skill development. In addition to academic and professional qualifications, selected contestants should also exhibit strong psychological resilience and possess key soft skills such as adaptability, teamwork, self-confidence, and a competitive spirit. These attributes ensure that participants have both a solid professional foundation and the comprehensive qualities needed to excel in the competition and effectively manage its challenges.

*“The scores from the final exam can serve as a reference point for selecting students”* (Student 11).

*“It is advisable to conduct early identification during the sophomore and junior years to recognize more capable*,* thoughtful*,* and motivated students for advanced training.”* (Student 14).

*“Contestants may be selected from students who have opted out of graduate school*,* secured their exam certainty*,* or committed to employment contracts. Their lack of concerns allows for undivided dedication to training.”* (Student 15).

*“In the selection of contestants*,* the primary focus should be on psychological resilience*,* adaptability*,* and teamwork. It is essential to identify students who prioritize teamwork over individual accomplishments within the group.”* (Student 3).

*“Confidence emerges as the most critical factor*,* highlighting the necessity for precise execution and clear thinking.”* (Student 6).

*“Being competitive*,* aggressive*,* and proactive in approach is vital for success in the competition.”* (Student 13).

## Discussion

### Optimizing training schedules for enhancing program feasibility and efficiency

This study, conducted through semistructured interviews with participating students, identified several areas for improvement in the competition training programs offered by nursing institutions. The findings revealed a need for enhancements in content and scheduling, training intensity, methodologies, and the establishment of operational standards. These deficiencies have led to issues such as overload, inadequate rest, and insufficient time for reflective training, ultimately contributing to inefficient and ineffective training outcomes. The development of scientifically robust pre-competition training programs in undergraduate nursing institutions is emphasized as essential for achieving optimal results in nursing technical skills competitions [22, 23]. To optimize training programs, participating students recommended the formulation of clear objectives and plans for different training periods, prioritization of training focus, and a progressive development of students’ abilities. Extending the training period was suggested to facilitate a more reasonable arrangement of content and effective responses to exceptional circumstances, thereby avoiding fatigue training that compromises efficiency. Furthermore, integrating video materials into the standard training process for enhanced learning and practice aligns with a strategy akin to Huang Qingying’s ‘four-step’ approach in secondary nursing skills competition selection and training [24], supporting the feasibility and rationale behind the students’ suggestions. Additionally, Zhang Shaomin’s emphasis on incorporating operational skills, humanistic qualities, psychological attributes, and critical thinking skills in training program development highlights the importance of a comprehensive approach [25]. Therefore, nursing colleges should be encouraged to lead training programs that systematically arrange content and scheduling, clarify methodologies, establish operational standards, and adjust training intensity according to competition requirements and components, ensuring the feasibility and scientific validity of the programs.

### Emphasis on mental health management and psychological support strategies during training

Our findings indicate that participants experienced negative emotions and faced challenges in coping with stress, both directly and indirectly, during the training process. These challenges manifested as difficulties in balancing training with ongoing educational commitments and job search activities, increased psychological stress, diminished self-confidence, and the exchange of negative emotions among team members. Collectively, these factors hindered participants’ ability to concentrate on training, reduced their resilience, and fostered an unfavorable team atmosphere, thereby adversely affecting the effectiveness and efficiency of the training. These results are consistent with Juanjuan Wang’s study, which found that 70.9% of participants reported experiencing high psychological stress during competition training [26]. In situations where participants, particularly when confronted with unfamiliar instruments, equipment, and operational objects at the competition site, struggle to effectively manage pressure, they may experience nervousness, anxiety, rapid heartbeat, disturbed thinking, decreased concentration, and transient brain amnesia. Such conditions can compromise their self-rational thinking and judgment, ultimately resulting in suboptimal performance at the assessment site [27–30]. Mei et al. [31] posited that nursing colleges should actively address the life, study, and work challenges faced by students, drawing from experiences in clinical skills competition training for college students. This approach aims to alleviate concerns and enable focused commitment to competition training. If contestants remain unable to mitigate psychological stress or struggle with coping strategies, the involvement of psychology professionals is recommended. These professionals can provide psychological adjustment and intervention counseling, assisting contestants in managing the stress and pressure associated with the competition and stabilizing their training level [32]. Furthermore, research indicates that an individual’s emotional intelligence and mental toughness can effectively predict their ability to cope with stress [33]. Therefore, nursing schools are encouraged, during participant selection, to not only assess students’ life, academic, and work challenges but also to evaluate their levels of emotional intelligence and mental toughness, ensuring the selection of the most suitable participants.

### Forming a high-quality competition training and nursing teaching team

The China Student Medical Technology Skills Competition serves as an assessment not only of the overall abilities of participating students but also as a measure of the competence of training instructors. Strengthening the training faculty and establishing a high-quality, highly qualified faculty team is essential. In the present study, participating students recognized the capability of training teachers to guide them, while emphasizing the importance of teachers’ willingness, availability of time, and energy for skill competition training. Furthermore, the personality and communication skills of training teachers were identified as factors influencing the team atmosphere and students’ psychological well-being. This observation aligns with Li et al.‘s recommendation to evaluate teachers based on their teaching ability and experience, while also considering their personality, family situation, strengths, weaknesses, and dedication [34]. The introduction of a ‘double tutor’ system, as discussed in Jiang Yan et al.‘s study, which involves collaborative training by faculty and clinical instructors, supports a cooperative approach to enhancing students’ fundamental knowledge, basic skills, and comprehensive abilities [35]. Additionally, involving physical education teachers and psychological professionals within the faculty is beneficial for ensuring students’ physical and mental well-being during training [35, 36]. Therefore, a thorough evaluation of faculty members, taking into account their suitability for training tasks, is critical to ensuring optimal performance by participating students.

### Enhancing and developing students’ comprehensive professional abilities through simulated the nursing skills competitions

The primary objective of the competition is to foster learning within a competitive framework. Consequently, the competition is structured with various components, including the formulation of questions, question types, and assessment formats, all designed to evaluate participants’ proficiency in fundamental knowledge, basic skills, and clinical thinking. Additionally, the competition assesses participants’ capabilities in teamwork and humanistic care [11]. Essentially, the competition is meticulously crafted to gauge participants’ comprehensive abilities, encompassing fundamental knowledge, basic skills, clinical thinking, teamwork, and humanistic care. However, this study found that participants perceived deficiencies in their comprehensive abilities during training, particularly in theoretical knowledge, operational foundation, humanistic care, clinical thinking, and the balance between operational speed and detail control. Similar findings in Jing Liu et al.‘s [37] study underscored the necessity of addressing irregular basic operation skills, enhancing clinical thinking training, improving teamwork abilities, fostering humanistic care, and avoiding the prioritization of operational speed over quality. The study suggests that achieving a qualitative leap in participants’ comprehensive abilities within a few months is challenging. Therefore, nursing colleges could integrate simulations of nursing technical skills competitions into the regular teaching process, adopting innovative teaching modes to explore long-term talent training mechanisms, thus ensuring a gradual and progressive enhancement of nursing undergraduates’ comprehensive abilities.

### Continued relevance and future research directions

Although this study is based on data collected from the 2021 competition, its findings remain relevant to current nursing education and skills competitions. Strategies such as optimizing training programs, prioritizing mental health, and establishing high-quality training teams may be applicable across various cultural contexts, although their implementation may require adjustments to align with different educational systems. The study’s findings are consistent with global trends in nursing education, which emphasize comprehensive competency development, the integration of practical and theoretical knowledge, and the importance of student mental health. This suggests that nursing education reforms in China are in line with international developments, and future research could investigate how these trends can be incorporated into nursing curricula worldwide. Furthermore, the educational value of skills competitions warrants further exploration in other countries, particularly with respect to enhancing practical skills, clinical reasoning, and teamwork. Expanding international collaboration through joint programs, competitions, and exchanges could strengthen global nursing education; however, challenges such as systemic differences and cultural adaptation may arise. Future research should address these challenges to provide valuable insights for global reforms.

### Limitations

Several limitations of the present study should be acknowledged. Firstly, we selected participants exclusively from two undergraduate nursing schools using an intentional sampling method, which may restrict the variability of the selected samples. Future research should aim to include students from undergraduate nursing programs across diverse regions and educational levels to enhance the representativeness of the sample. Secondly, as data were collected through post-competition interviews, recall bias may have influenced the participants’ responses. Conducting interviews at multiple time points throughout the competition process could provide more comprehensive and accurate insights. Lastly, since data collection occurred in 2021, it is possible that the experiences and perspectives of nursing students have evolved due to changes in healthcare education and practice. Future studies could address this limitation by employing longitudinal research designs or by comparing data across different years to track trends in nursing education and competition preparation.

## Conclusion

Nursing technical skill competitions have proven effective in enhancing both learning and teaching within the nursing field. Nursing colleges advocate for student participation in these competitions not only to achieve superior results, but also to identify challenges and shortcomings in nursing and clinical instruction. The findings emphasize the importance of nursing schools addressing the challenges, support needs, and suggestions of nursing students during the competition preparation process. It is crucial to establish a well-organized training schedule, address negative emotions, and provide timely psychological support to enhance training effectiveness. Additionally, attention should be given to the development of a high-quality, high-level competition training and nursing undergraduate teaching team. Furthermore, the implementation of comprehensive reforms in nursing education teaching methods is imperative for improving the professional competence of student interns, particularly in enhancing their clinical decision-making skills for analyzing and resolving nursing issues in specific clinical environments. These insights can assist nursing institutions in effectively designing training programs for nursing skills competitions and inform reforms in nursing teaching practices, ultimately bridging the gap between undergraduate nursing education and real-world clinical practice.

## Electronic supplementary material

Below is the link to the electronic supplementary material.

Supplementary Material 1

## Data Availability

The datasets used in the current study are available from the corresponding author on reasonable request. The data are not publicly available due to privacy or ethical restrictions.
