# Supplementary material for: Challenges and coping strategies of nursing interns in training for nationwide medical technical skills competition: a qualitative descriptive study
Source: BMC Nurs. 2025 Jul 1;24:730. doi: 10.1186/s12912-025-03387-0 (PMC12211161; doi:10.1186/s12912-025-03387-0)
Supplement: Supplementary file 1 — Supplementary Material 1 [file 12912_2025_3387_MOESM1_ESM.pdf]

Semi-structured Interview Guide: Exploring Nursing Students' Experiences and Training Improvement Suggestions in the National Medical Technical Skills Competition for College Students

Introduction

“Hello, thank you for taking the time to participate in this interview.

The purpose of this study is to gain insights into your experiences during the competition, exploring your participation motivation, personal growth, encountered challenges, and suggestions for improving training. This research will help us critically reflect on the limitations of our current practical teaching and ultimately enhance the quality of medical education.

This interview has two parts: first, a structured questionnaire about your general information which I will complete; second, a semi-structured interview exploring your competition experiences and suggestions for training improvement.

Your participation is crucial to our research. Please be assured that all your responses will be kept confidential, and your anonymity will be maintained. The interview is expected to last about 45 to 60 minutes. If you feel uncomfortable or don't want to answer any question, please let me know, and we can skip it or stop the interview.

Before we begin, may I have your permission to record this interview? This is solely to ensure we accurately capture your views.

Do you have any questions before we start? If not, I'd like to ask you some basic information firstly.”

I. General Information

1. Gender:

1☐ Male

2☐ Female

2. Age: ______ years

3. Family residence:

1☐ Urban area

2☐ Township

3☐ Rural area

4.The liberal arts and science stream division in Chinese secondary education*:

1☐ Liberal arts track

2☐ Science track

5. Occupational planning (Multiple choices allowed):

1☐ Nurse

2☐ Nursing teacher

3☐ Other professions related to nursing (e.g. medical representative)

4☐ non-nursing related occupations (e.g. civil service)

5☐ Other (please specify) _________

6. Previous experience of nursing skills competitionsl:

1☐ None

2☐ College-level competition

3☐ University-level competition

4☐ Municipal competition

5☐ Other (please specify) _________

7. Competition Registration:

1☐ Voluntary participation

2☐ College recommendation

3☐ Other (please specify)_________

8. Academic/Career status during competition preparation:

1☐ Recommended for postgraduate study

2☐ Preparing for postgraduate entrance exam (same institution)

3☐ Preparing for postgraduate entrance exam (different institution)

4☐ Engaged in job search

5☐ Other (please specify)_________

9. Type of award received in the Medical Technical Skills Competition： _________

____

** Note: In Chinese secondary education system, students typically choose between liberal arts track and science track, which influences their curriculum and university entrance examination*

II. Semi-structured Interview Guide

1.What was your motivation for participating in this competition?

2.What types of nursing skills competitions have you participated in before? How does this competition differ from your previous experiences? What specific gains did you achieve from this competition compared to previous ones?

3.What difficulties and challenges have you encountered during the training for the competition?

4.What kind of help or support do you expect in coping with the challenges in the training process?

5.What suggestions do you have to improve the feasibility and efficiency of preparation training for the competition?

6.How has participating in this competition influenced your professional identity and career development plans?

7.Please describe the most important personal and professional growth you experienced during the Competition.

8. Is there anything else about the competition experience that you would like to share, which we haven't discussed?

Closing

"Thank you very much for your time and valuable insights. Your contributions will greatly benefit our research. If you have any questions or concerns later, please don't hesitate to contact us."
